# Deep brain stimulation in post‐traumatic dystonia: A case series study

**DOI:** 10.1111/cns.13145

**Published:** 2019-04-29

**Authors:** Hong‐Xia Li, Lu He, Chen‐Cheng Zhang, Robert Eisinger, Yi‐Xin Pan, Tao Wang, Bo‐Min Sun, Yi‐Wen Wu, Dian‐You Li

**Affiliations:** ^1^ Department of Neurology & Institute of Neurology Ruijin Hospital, Affiliated with Shanghai Jiao Tong University School of Medicine Shanghai China; ^2^ Department of Functional Neurosurgery Ruijin Hospital, Affiliated with Shanghai Jiao Tong University School of Medicine Shanghai China; ^3^ Department of Neuroscience University of Florida Gainesville Florida; ^4^ Laboratory of Neurodegenerative Diseases & Key Laboratory of Stem Cell Biology, Institute of Health Science, Shanghai Institutes for Biological Sciences (SIBS) Chinese Academy of Sciences (CAS) & Shanghai Jiao Tong University School of Medicine Shanghai China

**Keywords:** deep brain stimulation, globus pallidus internus, post‐traumatic dystonia, subthalamic nucleus, ventral intermediate nucleus

## Abstract

**Aims:**

Deep brain stimulation (DBS) has been proposed as an effective treatment for drug‐intolerant isolated dystonia, but whether it is also efficacious for posttraumatic dystonia (PTD) is unknown. Reports are few in number and have reached controversial conclusions regarding the efficacy of DBS for PTD treatment. Here, we report a case series of five PTD patients with improved clinical benefit following DBS treatment.

**Methods:**

Five patients with disabling PTD underwent DBS therapy. The clinical outcomes were assessed with the Burke–Fahn–Marsden dystonia rating scale (BFMDRS) at baseline and the last follow‐up visit (at more than 12 months).

**Results:**

Patients 1 and 3 received unilateral globus pallidus internus (GPi) DBS for contralateral dystonia. The subthalamic nucleus (STN) was chosen as target for patients 2 and 4, due to a lesion located in the globus pallidus. Patient 5 had an electrode in the ventral intermediate nucleus (VIM) for treating predominant tremor of left upper extremity, with unexpected improvement of focal hand dystonia. The scores of BFMDRS movement exhibited favorable improvement in all five patients at the last follow‐up, ranging from 52.4% to 78.6%.

**Conclusions:**

Deep brain stimulation may be an effective and safe treatment for medically refractory PTD, but this needs to be confirmed by further studies.

## 1 INTRODUCTION

Post‐traumatic dystonia (PTD), the dystonia developing after brain injury, occurs in 4.1% of surviving patients.^1^ Hemi‐dystonia and focal‐hand dystonia are the most common forms of dystonia in PTD, while other forms of focal dystonia involving the neck, eyelids, and oral mandible, as well as segmental dystonia, multifocal dystonia, and generalized dystonia have also been reported.^2^, ^3^, ^4^ The lesions associated with PTD are usually found on Magnetic Resonance Imaging (MRI) or Computed Tomography (CT) of the brain, with the most common lesions located in the contralateral caudate, putamen, and thalamus.^5^ PTD may respond to common traditional dystonia medications. For instance, botulinum toxin injections are effective for focal dystonia. However, some PTD patients show low levels of satisfaction after botulinum toxin injection.^6^, ^7^ In recent years, deep brain stimulation (DBS) has become a suitable alternative for medication‐refractory isolated dystonia.^8^ Moreover, encouraging results of DBS treatment have been observed in tardive dystonia, a type of acquired dystonia.^9^, ^10^ However, only limited evidence supporting this treatment modality is available in patients with PTD, and exploratory results have been published mostly in the form of cases or single subjects in heterogeneous studies.^11^, ^12^ Here we report the clinical outcomes of our series of patients with disabling PTD who underwent DBS treatment. Furthermore, we review the extant literature on the treatment of PTD.

## 2 MATERIALS AND METHODS

This retrospective clinical study was approved by the ethics committees of the Ruijin Hospital Shanghai Jiaotong University School of Medicine. Each patient or his/her family agreed to participate in this study and provided their informed consent.

### 2.1 Patients

We recruited five patients undergoing DBS surgery at the Functional Neurosurgical Center of Shanghai Ruijin hospital from 2010 to 2017, who were diagnosed with PTD by a movement disorder neurologist. The inclusion criteria included: (a) history of brain trauma; (b) demonstrating PTD‐related difficulty in performing daily activities; (c) being intolerant of, or failing to respond to, common pharmacotherapy for dystonia (eg, levodopa, clonazepam, trihexyphenidyl, or baclofen) and botulinum toxin injection. The exclusion criteria were: (a) family history of dystonia; (b) dystonia resulting from other causes; (c) cognitive dysfunction; (d) other neuropsychiatric diagnoses.

### 2.2 Clinical evaluations

At baseline and at the last follow‐up visit (range 12 to 99 months), five patients were videotaped and evaluated by an experienced physician blinded to surgery status. The assessments involved dystonia severity, quality of life, and cognitive evaluations. The severity of the dystonia was quantified by the Burke–Fahn–Marsden Dystonia Rating Scale (BFMDRS), which consists of two components for assessing motor capability and the affected daily living. A higher BFMDRS score indicates greater impairment. Quality of life (QoL) was self‐evaluated by the Medical Outcomes Study 36‐item Short‐Form General Health Survey (SF‐36), which includes eight parts on general health, bodily pain, physical functioning, role‐physical functioning, vitality, social functioning, role‐emotional functioning, and mental health. A low SF‐36 score indicates a low quality of life. Cognitive function was evaluated by Mini‐Mental State Examination (MMSE). Both the SF‐36 and MMSE assessments have been recommended in dystonia for clinical research evaluation.^13^ For exclusion criteria, we considered impaired cognitive function to be MMSE scores of less than 24.

### 2.3 Surgical procedures

The globus pallidus internus (GPi), subthalamic nucleus (STN) and ventral intermediate nucleus (VIM) were selected as the location of surgery, which was decided by a multiple‐member disciplinary team. The locations were determined by using T2 weighted magnetic resonance imaging (MRI) (1.5 T; General Electric Healthcare) and confirmed with intraoperative macrostimulation. Quadripolar DBS electrodes (model 3387 for STN and GPi and 3389 for VIM; Medtronic) were implanted under local anesthesia, and motor function and adverse effects were strictly monitored during macrostimulation. The detailed location of GPi and STN target were mentioned in our previous study.^14^ VIM target planning started with standard stereotactic coordinates relative to the posterior commissure (PC) on the anterior–posterior commissure (AC‐PC)‐aligned MRI: 10.5‐11 mm lateral to the wall of the third ventricle, 6‐7 mm anterior, and 0 mm dorsal. Patients remained in our hospital for three nights using a 3625 external stimulator for trial stimulation. Permanent IPGs were implanted only after temporary stimulation showed preliminary efficacy. The IPG (Soletra, 37603 or 37612RC; Medtronic) was then implanted subclavicularly under general anesthesia. Postoperative MRI or CT was performed for each patient to confirm the location of the electrodes.

### 2.4 DBS programming

The IPG was turned on one day after implantation. DBS parameters were readjusted from one month after surgery when the local edema disappeared. We chose the most favorable parameters based on the most satisfactory improvement with the fewest stimulation‐related side effects, such as paresthesia and dysarthria.

### 2.5 Statistical analysis

QoL and MMSE scores at baseline and the last follow‐up visit were compared using a two‐tailed Mann–Whitney test. Results are expressed as means ± standard deviation (SD). Statistical analysis was performed using GraphPad Prism 6.0 (GraphPad Software). A *P*‐value of <0.05 was considered statistically significant.

## 3 RESULTS

### 3.1 Clinical characteristics and outcomes

The clinical characteristics of the five participants (one female and four males) are shown in Table 1. The patients received surgery at an age varying from 19 to 41 years. All patients were refractory to common medicines for treating dystonia, and several patients failed to respond to botulinum toxin injection.

**Table 1:** Summary of patient demographic data and clinical characteristics

ID	Sex	Age at trauma (years)	Loss of consciousness (Y,N/duration）	MRI lesions	Age at surgery (years)	History of dystonia	Latency of dystonia onset (months)	Dystonia type	Affected region	Persistence during sleep	Accompanying symptom (pain)	Failed treatment of medicine	Response to Botulinum toxin
1	M	7	Y/4 mo	Right midbrain	19	N	4	Focal dystonia	L‐upper extremity	N	Y	Mecobalamin, levodopa, clonazepam and trihexyphenidyl	Unknown
2	M	17	Y/6 d	Left globus pallidus	24	N	6	Hemi‐dystonia	R‐ upper extremity and R‐lower distal extremity	N	Y	Baclofen and trihexyphenidyl	N
3	F	28	Y/2 d	Bilateral external capsule	38	N	12	Generalized dystonia	L‐upper extremity, mouth, neck and L‐lower extremity	N	Y	Levodopa, clonazepam and trihexyphenidyl	Unknown
4	M	6	Y/2 h	Bilateral globus pallidus and putamen	34	N	6	Generalized dystonia	L and R upper distal extremities and trunk	N	N	Clonazepam and baclofen	Unknown
5	M	39	Y/1 mo	Right midbrain	41	N	18	Focal dystonia	L‐upper distal extremity	N	N	Levodopa, haloperidol, clonazepam and trihexyphenidyl	Unknown

Abbreviations: ID, patient identification number; sex: M, male; F, female; Affected region: L, left; R, right; Loss of consciousness: mo, months; d, days; h, hours; Y, yes; N, no.

**Figure 1:**
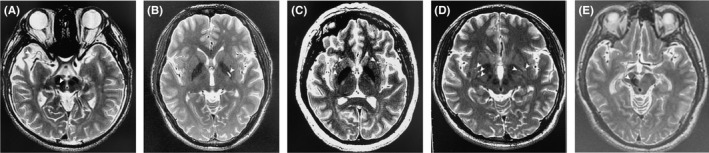
Preoperative brain MR images (T2‐weighted sequence) showing structure changes in the right midbrain and temporal lobe (A), the left globus pallidus (GP) (B), the bilateral external capsule (C), the bilateral globus pallidus (GP) and putamen (D), and the right midbrain (E). The location of the lesion is marked by a white arrow in each patient

Patient 1 suffered a severe closed head injury in a motor vehicle accident at the age of seven years. He was comatose for four months. He displayed a mild left hemiparesis after recovering from the coma. In transition to adulthood, the patient developed involuntary dystonic movement of the left upper extremity in lieu of the hemiparesis. The disability, focal dystonia of the left arm with remarkable spasticity and pain, severely exacerbated his function. A right midbrain lesion and an encephalomalatic change in the right temporal lobe, which resulted from the removal of hemorrhagic lesion, was evident on MR imaging (Figure 1A). Patient 1 had an electrode placed in the GPi contralateral to the dystonia.

Patient 2 was involved in a motor vehicle accident and sustained a traumatic brain injury at the age of 17. Six months after the initial head injury, a focal dystonia of the right upper extremity developed. Four months later, the sustained dystonia involved his right foot. The patient stated intolerable pain and cramp of his right foot, especially during walking. The dystonia was continuously present and affected his walking and balance. An MRI scan revealed lesion of the left globus pallidus (GP) before surgery (Figure 1B). Patient 2 received unilateral STN DBS.

Patient 3 sustained a closed head injury in a car accident at the age of 28 years. She reported difficulty in walking 12 months after the head injury, due to focal dystonia in the left lower extremity. Initially, the dystonia responded mildly to medical treatment. Five months later, she developed dystonia in the left upper extremity with severe spasticity and pain. The symptoms progressively involved the mouth and neck, but this did not bother her. A lesion of the bilateral external capsule was observed in the pre‐operative brain MRI (Figure 1C). Patient 3 underwent unilateral GPi DBS for the dystonic disability affecting her limbs on the left side.

Patient 4 experienced stiffness in his right thumb and wrist while writing six months after a fall caused injury to the patient's head and back at six years of age. By the age of seven years, he had started using his left hand for writing. However, the symptoms involved his complete left hand several months later. He could barely handle a pen due to the progression of the condition. At the age of 33 years, he developed dystonia that affected the trunk, especially during walking. A brain MRI showed abnormal signs in the bilateral GP and putamen (Figure 1D). Patient 4 received bilateral STN DBS.

Patient 5 suffered a severe closed brain injury in a car accident at the age of 39, leaving him in a coma for one month. He initially presented with left hemiparesis. Eighteen months after the accident, he developed resting and kinetic tremor of the left upper extremity with mild‐moderate dystonic hand posture. He was treated with unilateral VIM DBS for more severe tremor. Brain MRIs revealed a lesion of the right midbrain before surgery (Figure 1E).

The BFMDRS movement score of the five patients improved by 65.9% (range from 52.4% to 78.6%) and the disability score by 68.6% (range from 50.0% to 76.5%) (Table 2). The pain in the affected region also improved significantly in patients 1, 2, and 3, and patient 5 had a near complete tremor reduction. Moreover, all patients showed a remarkable improvement in quality of life evaluated by SF‐36 at the last follow‐up visit (Table 3). The cognitive function also remained on baseline at long‐term follow‐ups (Table 3). The stimulation parameters at the last follow‐up visit are presented in Table 2.

**Table 2:** Clinical outcomes of DBS

ID	Follow‐up (months)	Target	BFMDRS before DBS (movement/disability)	BFMDRS at last evaluation (movement/disability)	BFMDRS Improvement % (movement/disability/total)	Stimulation parameters of the last follow‐up visit [Amplitude(V)/ Frequency (Hz)/pulse width (us)]
1	14	R‐GPi	12/4	4/2	66.7/50.0/62.5	Right:2.65/170/90 Case(+) 1 (−) 2 (−)
2	12	L‐STN	28/17	6/4	78.6/76.5/77.8	Left:3.3/160/70 Case(+) 2 (−) 3 (−)
3	30	R‐GPi	11.5/4	4/1	65.2/75.0/67.7	Right:3.15/145/90 Case(+) 1 (−) 2(−)
4	24	B‐STN	21/9	10/3	52.4/66.7/56.7	Left:2.15/125/60 Case(+) 10 (−) 3.15/125/90 Case(+) 11 (−) Right:2.15/125/60 Case(+) 2 (−)
5	99	R‐VIM	6/4	2/1	66.7/75.0/70.0	Right:2.5/160/90 Case(+) 1 (−) 2(‐)

Abbreviations: ID, patient identification number; DBS, deep brain stimulation; BFMDRS, Burke–Fahn–Marsden Dystonia Rating Scale; Target: L, left; R, right; GPi, globus pallidus internus; STN, subthalamic nucleus; VIM, ventral intermediate nucleus; V, Volts; Hz, Hertz; μs, microsecond.

**Table 3:** Health‐related quality‐of‐life subscores on the Medical Outcomes Study 36‐item Short‐Form General Health Survey and Mini‐Mental State Examination at baseline and the last follow‐up visit

	Baseline scores (mean ± SD)	Last follow‐up scores (mean ± SD)	*P* value^a^
SF‐36 subscale			
General Health	38.0 ± 2.7	65.0 ± 7.6	0.008^b^
Physical Functioning	61.0 ± 20.4	95.0 ± 3.5	0.008^b^
Role‐Physical	20.0 ± 11.2	55.0 ± 20.9	0.04^b^
Vitality	45.0 ± 9.4	67.0 ± 8.4	0.02^b^
Social Functioning	47.5 ± 10.5	75.0 ± 12.5	0.02^b^
Role‐Emotional	40.0 ± 27.9	100.0 ± 0.0	0.008^b^
Mental Health	41.6 ± 9.2	58.4 ± 3.6	0.03^b^
MMSE	26.2 ± 2.2	26.8 ± 2.5	0.56^b^

Abbreviations: SF‐36, Health‐related quality of life sub‐score; MMSE, Mini‐Mental State Examination; mean ± SD, means ± standard deviation; score 0‐100.

a *P*‐values for comparisons between baseline and the last follow‐up.

b *P*‐value based on two‐tailed Wilcoxon signed rank (Mann–Whitney) test

### 3.2 Adverse events

Overall, the surgical procedures were well tolerated. There were no hardware‐related side effects, intracranial hemorrhages, infections, and lead or extension fractures from DBS implantation in the five patients within the 12‐month follow‐up. Dysarthria was elicited in patient 5, which was ascribed to stimulation intensities above the therapeutic threshold and improved immediately with reprogramming. Paresthesia was common in all patients when the parameters of stimulation were reset. The common adverse events of STN‐DBS in Parkinson's disease, fatigue and dyskinesia, were not observed in patients 2 and 4.

## 4 DISCUSSION

Post‐traumatic dystonia is one of the common movement disorders due to head trauma. In this retrospective study we reported the clinical outcomes of a series of PTD patients undergoing DBS treatment. In this cohort, we observed 56.7%‐77.8% improvement using BFMDRS in dystonia severity and disability in five patients. The accompanying symptom of body pain showed favorable improvement upon treatment with DBS in three patients. This is a clinically significant outcome since most patients suffering from PTD tend to be medically refractory. Also, it adds optional treatment for the same subgroup of acquired dystonia resulting from brain stroke.

Previous reports about the efficacy of DBS for treating PTD patients are few in number. The relevant case reports and studies are listed in Table 4. Most cases showed a favorable response to DBS, but there were several patients showing poor improvement. Kim et al^11^ reported that four patients with PTD undergoing GPi‐DBS showed a mean improvement on movement scores of BFMDRS of 73.2% at two‐year follow‐up. In our study, all patients showed a similarly favorable improvement. Compared to existing reports, our study is the largest case series describing preliminary clinical efficacy and safety in PTD as a result of DBS.

**Table 4:** Reviews of DBS for treating PTD

Reference	Number of patient	Age at trauma	Age at surgery	Latency of dystonia onset (mo)	MRI/CT lesions	Type of dystonia	Site of DBS	Degree of improvement	Follow‐up (mo)	Adverse effect	Stimulation parameters of the last follow‐up visit Amplitude/Frequency/Pulse width
Sellal et al^12^	1	16	16	Few	Anterior lateral region of the left thalamus	Hemi‐dystonia	L thalamus	Dramatic improvement of dystonic postures and movement of the upper right limb	8	Lesion of the scalp	Not mentioned
Loher et al^16^	1	15	18	Not mentioned	Subcortical and corti cal lesion of the left‐sided Frontal cortex	Hemi‐dystonia	R GPi	Remarkable improvement of dystonia‐associated pain, phasic dystonic movements, and dystonic posture	48	No	1V/130 Hz/150 μs
Chang et al^6^	1	17	23	36	Left GPi	Focal dystonia	L GPi	Significant improvement of cervical dystonia	12	No	2 V 160 Hz/ 180 µs/(initial parameters)
Slow et al^17^	1	5	26	Not mentioned	Right GPi	Focal dystonia	R GPi R thalamus	37% (movement) using BFMDRS improved; 44% using AIMS improved	48	No	R‐GPi 0−1−2+3.9 V/135 Hz/210 μs R‐Vim 3−0+3.6 V/135 Hz/90 μs
Zhang et al^25^	1	17	21	36	Thalamus	Multifocal dystonia	B STN	90.8% (total) using BFMDRS improved	24	No	Not mentioned
Martinez et al^26^	1	Not mentioned	Not mentioned	Not mentioned	No lesion	Generalized dystonia	L GPi	18.7% (movement) using BFMDRS improved pain reduction	Not mentioned	No	Not mentioned
Kwon et al^27^	1	3	47	Not mentioned	Cortex in the left parieto occipital region,Lcerebral peduncle, STN, thalamus, and parietal lobe	Hemi‐dystonia	L GPi	20% (movement) using BFMDRS improved; 50% (pain) using visual analog scale improved	60	No	Case +, contact 2−, 1.3 V/130 Hz/60 μs
Kang et al^28^	1	26	30	12	Left basal ganglia	Hemi‐dystonia	L GPi	85.7% (movement) using BFMDRS improved	24	No	Contact 0, 2.8 V/95 Hz/180 μs
Kim et al^11^	4	3, 12, 3, 37	23, 32, 26, 40	2, 13, 1, 9 mo	GP,putamen, Peritrigone, GP, GP	Hemi‐dystonia	L GPi	38.1%/66.7%, 75.0%/60.0%, 85.7% /75.0%, 94.1%/100% (movement/disability) using BFMDRS improved	30,70, 31,51	No	3.05 V/78.5 Hz/180 μs (mean parameters)
Margolesky et al^7^	1	5	30	Not mentioned	Gliotic changes in the GP and ventral thalamus as well as cystic encephalomalacia involving the right basal ganglia and corona radiata	Hemi‐dystonia	L STN	62.5% (total) using BFMDRS improved	9	No	Contact 9+10+, 2.6 V/80 Hz/70 μs
Ren et al^15^	1	30	50	324	No lesion	Focal dystonia	L GPi	64%/75%/66.7% (movement/disability/total) using BFMDRS improved	6	No	Not mentioned
Carvalho et al^29^	1	14	18	3	Right posterior thalamus	Dystonic tremor/Focal dystonia	R GPi	Substantial tremor suppression	48	No	Contact 3− Case +, 3.0 V/185 Hz/180 μs
Rojas‐Medina et al^21^	1	5	16	Not mentioned	Frontal, parietal and temporal bilateral leukomalacia	Intention tremor/Focal dystonia	L Vim	Marked improvement in the tremor and satisfactory improvement of his dystonia	96	No	3.4 V/130 Hz/120 μs

Abbreviations: PTD, post‐traumatic dystonia; L, left; R, right; B, bilateral; GP, globus pallidus; GPi, globus pallidus internus; STN, subthalamic nucleus; VIM, ventral intermediate nucleus; DBS, deep brain stimulation; BFMDRS, Burke‐Fahn‐Marsden Dystonia Rating Scale; AIMS, abnormal involuntary movement scale; V, Volts; Hz, Hertz; μs, microsecond.

The basal ganglia circuits were preserved in patients 1 and 3. Previous studies^15^, ^16^ showed that the GPi–DBS benefited significantly from treating focal dystonia acquired as a result of brain trauma. Thus, we selected the GPi as the site of stimulation in patients 1 and 3. As is known, isolated lesions of the GPi can result in dystonia in healthy people,^6^, ^17^ but in patients with dystonia, stimulation or lesioning of the same structure can ameliorate this condition. This observation indicates that a disturbed pallidal discharge and abnormal output of the pallidothalamus would be responsible for acquired dystonia.^18^ In our study, patients 2 and 4 had abnormal GPs on MRI images. The reported studies^19^, ^20^ indicated that STN DBS was efficacious in treating dystonia in patients who already underwent bilateral pallidotomy. Thus, we placed an electrode in the dorsal STN. Patient 5 had resting and kinetic tremor of the left upper extremity. VIM was selected as the target for the disabling tremor, but surprisingly we found that stimulation of VIM also attenuated the focal hand dystonia. Besides, some case reports^21^, ^22^ also indicated that VIM DBS resulted in improved dystonia as well as tremor reduction.

Currently, there is uncertainty regarding the optimal lead location due to brain lesions existing in patients with PTD. There was no significant difference in degree of improvement between STN and GPi DBS in treating isolated dystonia with no brain lesion, as indicated by our previous study. However, the study by Margolesky et al^7^ showed that stimulation of STN could be given as a rescue therapy for PTD with a structurally abnormal GP tolerant to GPi‐DBS. Our data also show that patients with lesion of the GP area have a favorable response to STN‐DBS. The lenticular fasciculus (LF) courses, which are dorsal to the STN, are composed of fibers from the GPi and carry motor signals from the basal ganglia to the thalamus.^23^ STN projection in the indirect pathway and GPi fibers of passage (LF courses) represent possible therapeutic targets of DBS in the STN region.^24^ Then, the possible remaining functional neural circuitry allows for a response to the input of STN DBS, when there was an abnormal GP structure causing the dystonia. Moreover, stimulation in the dorsal part of the STN bypasses the GPi en route by activating LF courses to the thalamus and subsequent motor outflow tracts. These results indicate that STN may be the optimal target in treating PTD with a lesion of the GP area.

As the mechanism underlying the effects of deep brain stimulation (DBS) procedures are not well understood, we also followed the cognitive status. During the long‐term follow‐up, DBS had no noticeable influence on patients' perception in our report, which mostly agreed with studies of DBS on isolated dystonia.

The small number of patients tested was a limitation to our study (n = 5). A larger sample population is likely to result in more objective and credible outcomes. Also, the heterogeneity between patients would be a concern for further studies, even if PTD is not a common condition. Further studies may explore the optimal target for surgical treatment of PTD.

## 5 CONCLUSIONS

Based on the limited information available, DBS may be a potential treatment for medically refractory PTD, but this needs further exploration. Furthermore, to assess whether the GPi or the STN is preferable in PTD with GP lesion will require larger studies to reach definitive conclusions.

## CONFLICT OF INTEREST

Dr Dian‐you Li and Dr Chen‐cheng Zhang have received honoraria and travel expenses from the Deep Brain Stimulation industry (Medtronic, PINS, SceneRay). Dr Bo‐min Sun received research support from PINS and SceneRay (donated devices); The other authors have no conflicts.
